# The Use of the Velocity Selective Recording Technique to Reveal the Excitation Properties of the Ulnar Nerve in Pigs

**DOI:** 10.3390/s22010058

**Published:** 2021-12-23

**Authors:** Felipe Rettore Andreis, Benjamin Metcalfe, Taha Al Muhammadee Janjua, Winnie Jensen, Suzan Meijs, Thomas Gomes Nørgaard dos Santos Nielsen

**Affiliations:** 1 Center for Neuroplasticity and Pain (CNAP), Department of Health Science and Technology, Aalborg University, 9220 Aalborg, Denmark; taha@hst.aau.dk (T.A.M.J.); wj@hst.aau.dk (W.J.); smeijs@hst.aau.dk (S.M.); thnn@hst.aau.dk (T.G.N.d.S.N.); 2 Center for Biosensors, Bioelectronics and Biodevices (C3Bio), Department of Electronic & Electrical Engineering, University of Bath, Bath BA2 7AY, UK; b.w.metcalfe@bath.ac.uk

**Keywords:** compound action potential, electroneurography, velocity selective recording, multi-electrode cuff, neural recording

## Abstract

Decoding information from the peripheral nervous system via implantable neural interfaces remains a significant challenge, considerably limiting the advancement of neuromodulation and neuroprosthetic devices. The velocity selective recording (VSR) technique has been proposed to improve the classification of neural traffic by combining temporal and spatial information through a multi-electrode cuff (MEC). Therefore, this study investigates the feasibility of using the VSR technique to characterise fibre type based on the electrically evoked compound action potentials (eCAP) propagating along the ulnar nerve of pigs in vivo. A range of electrical stimulation parameters (amplitudes of 50 μA–10 mA and pulse durations of 100 μs, 500 μs, 1000 μs, and 5000 μs) was applied on a cutaneous and a motor branch of the ulnar nerve in nine Danish landrace pigs. Recordings were made with a 14 ring MEC and a delay-and-add algorithm was used to convert the eCAPs into the velocity domain. The results revealed two fibre populations propagating along the cutaneous branch of the ulnar nerve, with mean velocities of 55 m/s and 21 m/s, while only one dominant fibre population was found for the motor branch, with a mean velocity of 63 m/s. Because of its simplicity to provide information on the fibre selectivity and direction of propagation of nerve fibres, VSR can be implemented to advance the performance of the bidirectional control of neural prostheses and bioelectronic medicine applications.

## 1. Introduction

The idea of using electrical stimulation to modulate activity within the peripheral nervous system (PNS) dates to Luigi Galvani (1737–1798). The modulation of the PNS has since been investigated in a wide range of applications, including, but not limited to, restoring impaired sensory and motor functions [1]; the treatment of diseases, such as depression [2] and epilepsy [3]; the modulation of the heart [4]; and control of the urinary bladder [5]. The information contained in the PNS is rich, with fibres (axons) having properties according to their function: sensory, motor, or autonomic. Individual fibres can be further characterised by their diameters (related to the conduction velocity and fibre excitability), direction of propagation (i.e., afferent or efferent), and degree of myelination [6]. Such characterisations are directly related to function, meaning that different groups of fibres are responsible for conveying specific information to the central nervous system and controlling, for instance, skeletal or cardiac muscles [7]. However, because of practical challenges (such as the low signal amplitude and interference from extraneural sources), decoding the peripheral neural information remains a significant challenge, considerably limiting the clinical usability of peripheral neural interfaces [8,9].

Implantable neural interfaces can be classified into three main groups: intraneural, regenerative, and extraneural [10]. While intraneural and regenerative interfaces provide a better selectivity because of the smaller distance from the nerve fibres to the electrodes, their level of invasiveness increases the chance of inducing damage to the neural tissue, either directly or through inflammatory processes [10]. Still, recent studies have successfully used intraneural interfaces in chronic experiments (for a comprehensive review on implantable peripheral nerve interfaces, see [7], and for applications in sensory feedback for limb prosthesis, see, e.g., [11]). Extraneural interfaces are positioned outside the epineurium, being less invasive and, thus, preferable for chronic implantation [12]; however, due to the greater distance between the electrodes and the individual fibres, they have both a lower signal-to-noise ratio (SNR) and lower selectivity [10].

The cuff electrode is the most well-established extraneural nerve interface. It has proven to be stable and safe for chronic implants and has been in clinical use for several decades [13,14]. Traditionally configured as a single bipole or tripole, signals recorded using cuffs include energy from active nerve fibres and, thus, have relatively low selectivity and SNR. Thus, the ability to discriminate between neural signals from different groups of fibres is limited. To improve the performance of recording using cuffs, Taylor et al. [15] proposed the velocity selective recording (VSR) technique, which combines spatial and temporal information to convert the time-domain signal into the velocity domain [16]. The VSR technique allows the discrimination of the signal based on the properties of its fibre-type populations (i.e., conduction velocity) and the direction of propagation (i.e., afferent or efferent). It also increases the SNR by $\sqrt{n}$, where *n* is the number of channels in the multi-electrode cuff (MEC) [17,18]. To date, the VSR has been investigated in animal models, including the rat dorsal root [19], frog sciatic nerve [20,21], earthworms [22], pig vagus nerve [23], and pig median nerve [24]. In these experiments, the effect of stimulation amplitude was considered; however, no investigation was performed to analyse the effect of the pulse duration on the recruitment of nerve fibres. The stimulus duration is an important parameter when probing the PNS, as it affects the threshold difference between fibres of different diameters [25] and the stimulation efficiency (i.e., minimise the energy required for neural excitation).

The pig as an animal model has a considerable degree of resemblance to human anatomy and physiology [26,27]. Porcine models are already established in several translational research areas, such as diabetes [28] and toxicology [29]. Pigs have also been proposed as suitable models for neuroscience research due in part to their brain similarity to humans [30]. For studies concerning the PNS (e.g., nerve repair and neural interfaces), pigs have the advantage that the fascicular organisation and overall morphology of the nerve are comparable to humans [31]. Thus, they can serve as better pre-clinical models for both the design of new interfaces and nerve repair models [32], as well as advancing therapeutic effects of neuromodulation [31].

In the PNS, the ulnar nerve is an important target when considering peripheral nerve injuries [33], sensory restoration [34,35], and motor control [36] due to its innervation of muscles and skin in the forearm and hand. In pigs, it is an easily accessible nerve that does not need the dissection of deeper structures, making it a promising target to investigate long-term implants [32] and chronic experiments, in which electrical stimulation is used to probe neural circuitry. Furthermore, along with the median nerve, the innervation region covers nearly the entirety of the palmar and finger areas, making it a valuable site for investigating the bidirectional control of hand and forearm prostheses [37,38]. Besides prosthetic applications, the ulnar nerve has significant clinical importance for investigating several neuromuscular disorders (e.g., polyneuropathy and carpal tunnel syndrome) [39]. In pigs, both the ulnar and median nerves have similar morphology to humans [40], and a preliminary application of the VSR technique has been demonstrated in the median nerve of a pig [24]. The authors found that two fibre populations were recruited, the first at 60 m/s and the second at 30 m/s with thresholds of 200 μA and 4 mA, respectively. However, in this study, the effect of pulse duration on the electrically evoked compound action potentials (eCAPs) was not investigated. To our knowledge, no investigation has ever been performed with the VSR technique on the ulnar nerve.

Therefore, in the present study, in vivo experiments on the ulnar nerve of pigs were performed to investigate the functional selectivity (i.e., the ability to record from specific fibre types) with the VSR technique. Secondly, the role that stimulus amplitude and pulse duration contribute to the excitation of the fibre populations within the ulnar nerve was explored. Section 2 introduces the materials and surgical methods, Section 3 presents the results with statistical analysis from nine pigs, Section 4 discusses the implications of the results, and, finally, Section 5 presents the conclusions.

## 2. Materials and Methods

### 2.1. Animal Preparation

All animal procedures were performed in accordance with the Danish Veterinary and Food Administration under the Ministry of Food, Agriculture and Fisheries of Denmark (protocol number 2017-15-0201-01317). Nine female Danish Landrace pigs, with a mean weight of 34.5 kg (range: 29.8–39.0 kg), were used for this study. The pigs were acclimatised 2 weeks before each experiment in a room with a temperature maintained at ~24 °C with a 13:11 h light–dark cycle. During the surgery, the pigs were intubated with a 1:1 oxygen and air mixture, and a constant infusion of isotonic saline was administered via the jugular vein. The animals were anesthetised with sevoflurane (1.5 to 2.5 % minimum alveolar concentration), propofol (2 mg/hr/kg), and fentanyl (10 ug/hr/kg). After implantation of the electrodes, propofol and fentanyl levels were increased by 100%, and sevoflurane was gradually decreased to 0. This was done to minimise the suppression of cortical response for another study not reported here. The animals were mechanically ventilated at 15 cycles per minute. The depth of anaesthesia was monitored through the body temperature, end-tidal CO_2_, oxygen saturation, heart rate, respiration rate, blood pressure, and tail reflex. The mean central body temperature was 37.6 ± 0.9 °C (between animal variability). Once the temperature was stable, it was maintained at this level during the experiment using a temperature-controlled air blanket (Mistral-Air Plus, MA1100-EU) placed under the pig. At the end of the experiment, the animals were euthanised by an overdose of pentobarbitone.

### 2.2. Electrodes & Instrumentation

**Figure 1:**
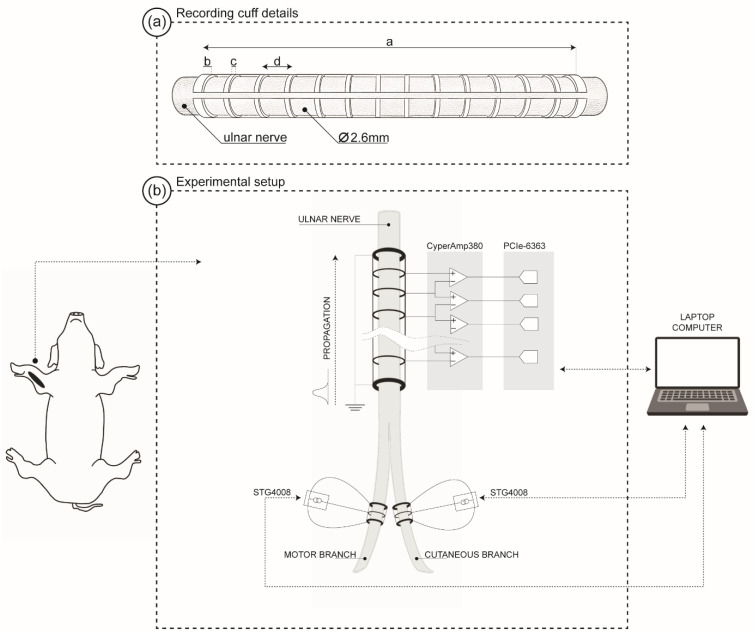
(**a**) Diagram of the 14 rings multi-electrode cuff (MEC). The length of the MEC (a) was 50 mm, with end-rings (b) of 1 mm width, the centre electrodes (c) had a width of 0.5 mm, and the inter-electrode distance (d) was 3.5 mm. The approximate diameter of the cuff was 2.6 mm. (**b**) Experimental setup for recording the neural responses evoked by ulnar nerve electrical stimulation. Tripolar cuffs were used for the stimulation, whereas a multi-electrode cuff was used to record the electroneurogram (ENG) signal.

Three cuff electrodes were custom-made in our laboratory, according to the technique described by Haugland [41]. Two simulation cuffs were used: one for the dorsal cutaneous branch of the ulnar nerve and another for the muscular branch (motor branch) of the ulnar nerve. The stimulation cuffs were 10 mm long, with an inner diameter of 1.8 mm, and contained 3 platinum-iridium ring electrodes with a 3 mm centre-to-centre distance. The end electrodes were 1 mm wide, and the centre electrode was 0.5 mm wide. Additionally, an external potentiometer was connected between the end electrodes and the stimulation return to balance the electrode impedances. Reducing the potential difference between the outer rings allows minimising the current flow past the recording system, significantly lessening stimulation artefact contamination [42]. The potentiometer was adjusted during 1 Hz continuous stimulation and visual inspection of the evoked response to a setting that minimised the magnitude of the artefact. The recording cuff (Figure 1a) was 50 mm long, with an inner diameter of 2.6 mm, and contained 14 ring electrodes: 12 centre electrodes with a width of 0.5 mm and 2 end electrodes of 1.0 mm width. The inter-electrode distance was 3.5 mm. The end electrodes of the recording cuff were short-circuited and used as a reference. The animal was grounded via a subcutaneous stainless steel probe connected to the epidermis and to the recording amplifier. For the implantation of the electrodes, an incision of approximately 20 cm was made on the anterior forearm to expose the ulnar nerve and its two branches. A section of approximately 15 cm of the ulnar nerve was freed from surrounding tissues. The cuff electrodes were then placed around the nerve by opening a slit in one end of the cuff, pushing the nerve through, and closing the cuff again. A silicon sheet was placed around the cuff to minimise current leakage, and the cuff was closed with ligatures at the ends and the centre.

The recording cuff was connected to an amplifier bank (CyberAmp 380, Axon Instrument Inc., Burlingame, CA, USA), with an amplifier input noise of 1.4 μV_rms_ over a 10 kHz bandwidth. The gain was chosen depending on the signal amplitude, and the mean amplifier voltage gain was 75 dB (range 66–86 dB). The signals were then bandpass filtered using a fourth-order Bessel filter with–3 dB frequency at 100 Hz and 10 kHz. The signals were sampled simultaneously at 90 kS/s with 16-bit resolution using a PCIe-6363 and a BNC-2090 connector (National Instruments, Austin, TX, USA). A representation of the experimental setup is shown in Figure 1b.

### 2.3. Experimental Protocol

A programmable stimulator (STG4008, Multichannel Systems, Reutlingen, Germany) was configured to produce trains of asymmetric rectangular charge-balanced biphasic pulses with amplitudes ranging from 50 μA to 10 mA in 4 different levels of pulse-width: 100 μs, 500 μs, 1 ms, and 5 ms. Based on a pilot experiment, different step sizes for the stimulation amplitude were used, with smaller step sizes in ranges in which the change in the recruitment was most significant (i.e., 50 μA steps from 0 to 1 mA), and larger step sizes (i.e., 1 mA steps from 7 mA to 10 mA) in which the stimulation was supra-maximal for the majority of the fibres. The secondary phase had an amplitude of 10% of the primary pulse. In 5 animals, the primary and secondary phases of the stimulation had a separation of 100 μs, while, for the rest of the animals, the delay was 33 ms. The long delay between the primary and secondary phase was chosen for a potential investigation of secondary pulse excitability, not reported here. The delay between each stimulation was 1 s with a pseudo-random Gaussian interval with a maximum of 250 ms. The delay was used to prevent stimulus-specific adaptation, as cortical signals were simultaneously recorded with the eCAPs (not reported in this study). The entire stimulation profile was repeated four times.

### 2.4. Data Analysis

The raw time-domain electroneurogram (ENG) was segmented based on the onset of the stimulus, using epochs from −1 ms pre-stimulus to 10 ms post-stimulus, and then bandpass filtered using an eighth order Butterworth filter with cut-off frequencies of 300 Hz and 8 kHz, respectively, as most of the ENG power lies between 1 and 3 kHz [9]. The eleven bipolar filtered signals were converted to ten tripolar signals by calculating the difference between pairs of adjacent channels, as the tripolar configuration has a better performance than the bipolar and monopolar configuration for rejecting common-mode interference [43], and DC offsets were removed. Then, the signals were analysed using the delay-and-add algorithm [15,20]. This algorithm operates by artificially delaying each channel relative to the first channel of the multi-electrode cuff. The delay is dependent both on the spacing between electrodes and conduction velocity; in the case of equal spacing between the electrodes, the second channel is delayed by dt, the third channel by 2 × dt, and so on. Thus, by using a range of delay values, the output signal will show a peak as the delay matches the quotient of the inter-electrode distance and the conduction velocity of the action potential. This procedure results in a transformation from the time-domain to a single velocity-domain signal, the intrinsic velocity spectrum (IVS) [18].

From the IVS, the amplitude peaks and their respective velocities were extracted. The amplitude threshold was set to five times the baseline standard deviation to avoid detecting false peaks (i.e., sharp changes in the IVS in the absence of propagating time-domain signals). Values that did not cross the threshold were considered as not activating the nerve, or the signal was contaminated by stimulation artefact.

Mathematical models can be used to provide a framework for characterising the eCAP with fewer data points, and characterise the recruitment curves with reduced parameters. Hence, three mathematical models were used to fit the eCAP growth functions, based on the peak value of the IVS and controlled for the animal, pulse-width, and stimulation site. The implemented models were: (1) four-parameter logistic model, (2) a four-parameter logistic model with the horizontal asymptote constrained to zero, and (3) the Gompertz function. To assess goodness-of-fit, the Akaike information criterion (AIC) was extracted for each model. A one-way repeated measures ANOVA was used to compare the AIC of the models. In the case of a significant difference, the model with the lowest AIC was implemented. A linear mixed model with Tukey’s multiple comparison test was used to assess the differences in the amplitude recruitment patterns for the four levels of pulse duration. The adopted level of significance was 0.05.

All data processing was performed offline using MATLAB 2020a (MathWorks, MA, USA) and R [44]. Graphical outputs were obtained using either MATLAB or ggplot2 [45].

## 3. Results

The ulnar nerve was easily identified and accessed in all the experiments, and dissection did not risk injury to neighbouring structures. In three animals, eCAPs could not be identified in one of the branches because of stimulation artefact contamination. This problem was worsened in the case for which the nerve was stimulated with longer pulse durations. For the rest of the animals, however, there was no stimulation artefact contamination in the eCAPs, even though the distance between stimulating and recording the electrodes was relatively short, at approximately 2.5 cm. Visual inspection showed a significant effect in reducing the stimulation artefact by balancing the impedances of the outer electrodes of the stimulation cuffs.

The SNRs were characterised in one animal, and the signals showed, over all channels, a mean noise floor of 4.3 µV_RMS_ (approximately 3 times the noise floor for the amplifiers) or, in peak-to-peak value, a noise floor of 13.5 µV_pp_. The first observable eCAP had a mean amplitude of 51.4 µV_pp_ when the nerve was stimulated with a stimulus amplitude of 350 µA and a pulse-width of 100 µs, resulting in an SNR(V_pp_/V_pp_) of 5.8 dB. At higher stimulation intensities, such as 10 mA, the mean amplitude of the eCAP was 532 µV_pp_, resulting in an SNR of 15.9 dB. Additionally, to verify the interface stability and safety, a comparison in the detected velocity for an eCAP elicited with the same stimulation parameters in the beginning and at the end of the experiment showed only a variation of 1.8%. Likewise, visual inspection did not reveal any signs of nerve injury.

**Figure 2:**
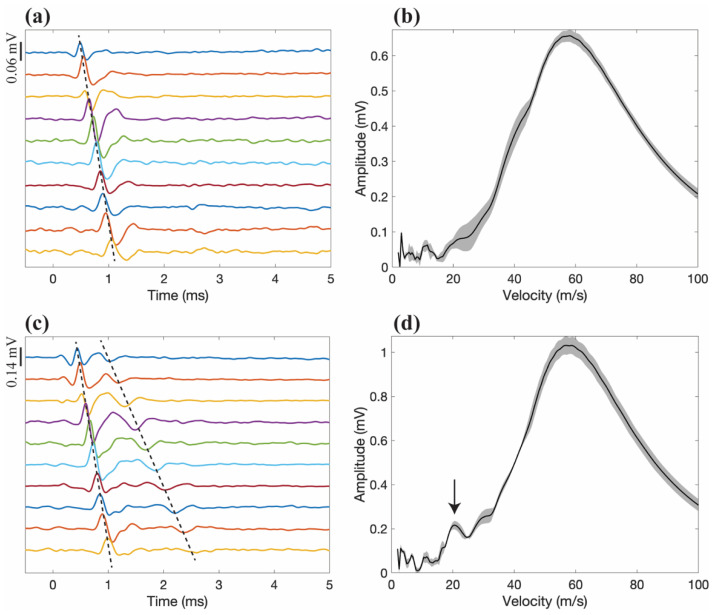
(**a**) Example of an electrically evoked compound action potential (eCAP) response recorded from the cutaneous branch with a stimulus of 350 μA and a pulse-width of 100 μs. (**b**) The corresponding intrinsic velocity spectrum (IVS) showing a peak velocity of around 58.5 m/s. (**c**) Responses recorded with a stimulus of 3.5 mA and a pulse duration of 100 μs. (**d**) The corresponding IVS, showing two peak velocities, one with approximately 58.5 m/s and the second with a velocity of roughly 22 m/s. From the IVSs, the shaded area represents the standard deviation of the four repetitions. In the time-domain eCAPs, offsets were artificially created for ease of visualisation.

Figure 2 is an exemplar illustration of the eCAP and the corresponding IVS resulting from stimulation at 350 μA (Figure 2a,b) and 3.5 mA (Figure 2c,d), with a pulse duration of 100 μs. Artificial offsets were added to the time-domain representation for visualisation purposes. The time-domain signals (Figure 2a,c) show the tripoles with a slight delay from each other, illustrating the propagation of the eCAP. The resultant IVS (Figure 2b,d) shows the resulting transformation after the delay-and-add algorithm.

Figure 2a shows an example with a single dominant conduction velocity, which can be seen in the corresponding IVS, shown in Figure 2b, with an apparent peak velocity of 58.5 m/s. Figure 2c shows an example in which two conduction velocities are present, and the corresponding is shown in IVS Figure 2d, with peaks at 58.5 m/s and 22 m/s. By manually estimating the conduction velocity from the time-domain signal (slope of dashed lines), the velocities were calculated to be 59 m/s and 21.5 m/s. While the eCAPs illustrate the recordings from only one stimulation event, the IVSs show the average and standard deviation of four stimuli with the same amplitude and pulse-width, showing that the IVS profile is maintained across repetitions.

### 3.1. Velocity Distribution

**Figure 3:**
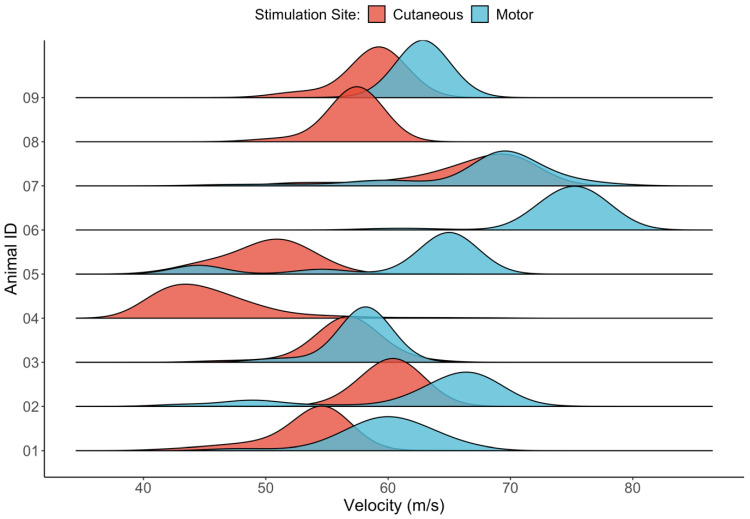
Density estimates of fast fibre (Aβ group), extracted from the intrinsic velocity spectrum (IVS) for each animal and stimulation site.

To characterise the range of velocities supported by the ulnar nerve, the dominant velocities (those for which a distinct peak was observable) were extracted from the IVS for the whole range of stimulation amplitude and levels of pulse duration. All animals displayed a dominant velocity within the expected range of the Aβ fibres [35–75 m/s]. Figure 3 shows the density estimates for the velocity of the fast fibres (Aβ group) for each animal and stimulation site (i.e., the stimulated branch). The effect of the pulse duration was not considered here, because this effect is expected to be reflected in the threshold of activation and not on the velocity distribution.

Figure 3 shows that the variability in the velocity distribution is mainly explained by inter-animal variation, having a narrower intra-animal distribution. In the cutaneous branch, the detected velocities varied from approximately 40 m/s (Animal 04) to 70 m/s (Animal 07), whereas most of the motor branches are in the velocity range between 60 m/s and 75 m/s. Additionally, the motor branch always displays a higher velocity than the cutaneous branch. A paired sample t-test showed the difference to be significant (*p* < 0.05). The normality distribution was verified through the Shapiro–Wilk test (*p* = 0.5). The mean velocity for each animal across all stimulation amplitudes and pulse durations is shown in Table 1.

Besides the inter-animal variability in the detected conduction velocity, intra-animal variability was also observed. Table 1 shows a standard deviation in the detected peak ranging from 1.1 m/s (Animal 09) to 8.4 m/s (Animal 07).

**Figure 4:**
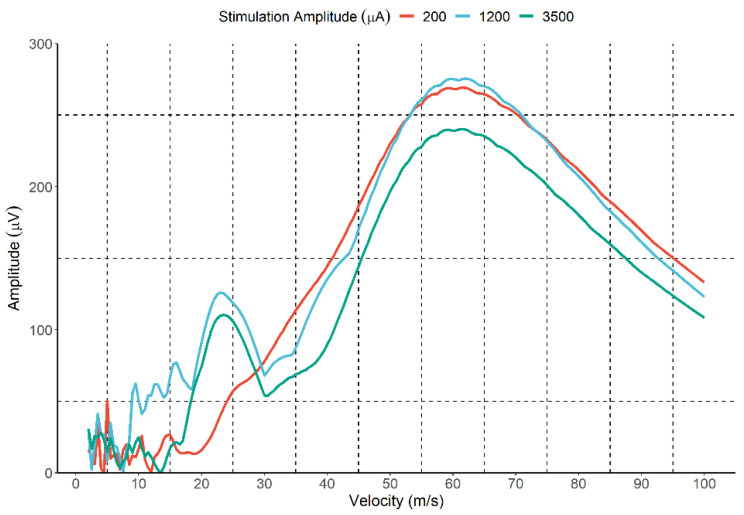
Excitation of fast and slow fibres by high stimulation intensity. In red, it is possible to see the activation of only the fast fibre group with a stimulation amplitude of 200 μA. At the intensity of 1200 μA (blue line), slow fibres are detected in the IVS, with a velocity of approximately 22 m/s. The IVS profile is maintained for the higher intensity of 3500 μA (green line).

Two fibre populations were detected for only three animals. In all the cases, it was found when the cutaneous branch was stimulated. For the slow-fibre population, animals had a mean velocity of 24.8 m/s, 20.4 m/s, and 18.1 m/s, with a within-animal standard deviation of 2.34 m/s, 4.72 m/s, and 4.26 m/s, respectively. The observed conduction velocity is consistent with the expected values for the Aδ fibre group [range 5–35 m/s]. An example of the IVS of an animal with two populations of fibres is presented in Figure 4. The figure shows the effect of increasing the stimulation intensity on the IVS, with three stimulation intensities (200 μA, 1200 μA, and 3500 μA).

At the lower intensity (200 μA), only fast fibres were activated, with a velocity of approximately 60 m/s. When increasing the stimulation (1200 μA and 3500 μA), two groups of fibres are recruited with different velocities (60 m/s and 22 m/s). Since two populations of fibres were only detected in three animals, the following Section explores the effect of the stimulation amplitude and pulse duration on the activation of the fast fibre population (range of velocities), which was observed for all the subjects.

### 3.2. Effect of Stimulation Amplitude and Pulse Duration

No significant difference was found between the AIC of the three models used to fit the amplitude recruitment curves (*p* = 0.94). Therefore, the Gompertz model was adopted for the following sections, which is frequently used for modelling recruitment curves as a response to electrical stimulation [46].

**Figure 5:**
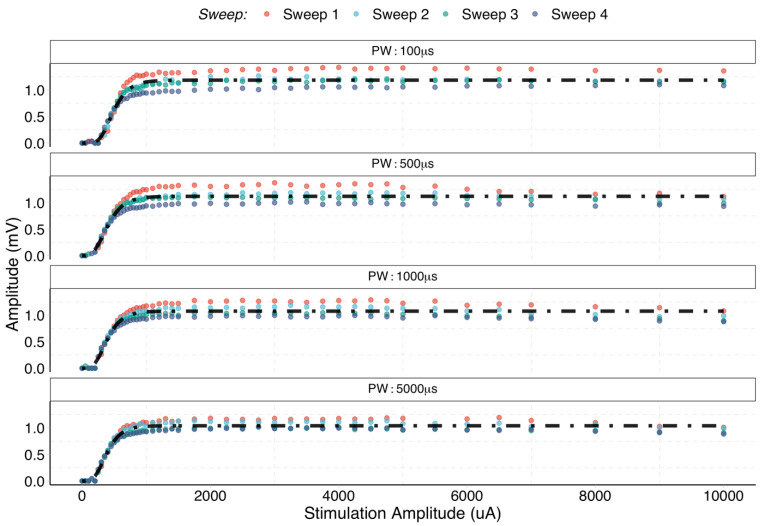
Observed (dots) and predicted values (dot–dashed line) for a single animal. Each colour represents a single eCAP from the four repetitions (i.e., sweeps) for each stimulation value and level of pulse-width.

The amplitude of the dominant velocity of the IVS as a function of stimulation amplitude and pulse-width from one experiment is shown in Figure 5. The individual observations (dots) are coloured by repetition (sweeps), and the fitted model is represented as a black dashed–dotted line. The fitted models show that the amplitude profile is similar for each pulse width. Moreover, there are no significant differences in the amplitude for each sweep, which reflects two important points: (1) it shows a stable interface for the duration of the experiments and (2) that the peak amplitude of the IVS is maintained across repetitions, for a given current and pulse-width. The threshold of activation of the fast fibre population (Figure 3) was found to be between 100 μA and 200 μA, for all animals and levels of pulse duration.

**Figure 6:**
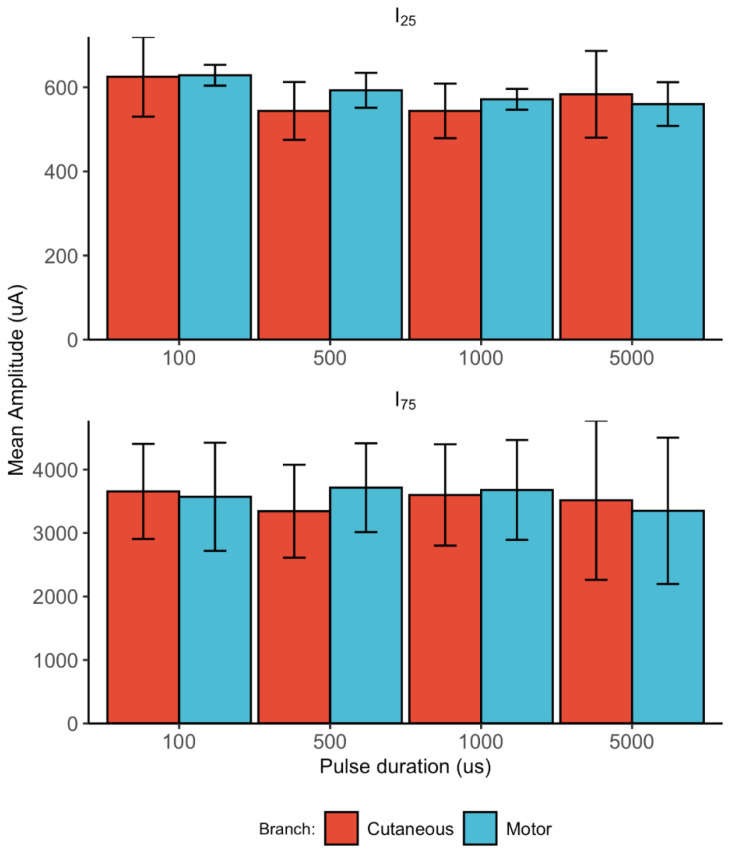
Mean amplitude to activate 25% (**top panel**, I_25_) and 75% (**bottom panel**, I_75_) of the nerve. The error bars represent the 95% confidence intervals.

Subsequently, to investigate the effect of the pulse duration on the amplitude recruitment patterns, the thresholds to produce 25% (I_25_) and 75% (I_75_) activation of the fast-fibre population were extracted. The results for the two branches and four values of pulse-width are shown in Figure 6.

There was a significant effect of the pulse duration to activate 25% of the nerve for the cutaneous (*p* = 0.01) and the motor (*p* < 0.01) branch of the ulnar nerve. For both the motor and cutaneous branches, the necessary current to excite 25% of the nerve was significantly higher for a pulse duration of 100 μs than for the other levels of pulse duration (i.e., 500 μs, 1000 μs, and 5000 μs). There was no significant difference in the pulse durations to activate 75% of the nerve for both the cutaneous (*p* = 0.06) and the motor (*p* = 0.45) branches.

## 4. Discussion

This paper has demonstrated the feasibility of using the VSR technique to characterise the excitation properties and distinguish fibre type in the ulnar nerve of pigs. The VSR technique allows differentiating between fibre types, according to their propagation velocity and direction of propagation (i.e., afferent or efferent). In the context of signals from upper and lower extremities, efferent signals can be used to activate prostheses and paralysed limbs, while decoding afferent signals into their specific functions (i.e., conduction velocities) can be used to provide sensory feedback for tactile and force sensors. The classification of peripheral nerve information based on fibre-type is also essential in neuroscience experiments, in which electrical stimulation is used to activate neural tissue. In this case, the VSR technique can be used as an online tool to assist in selecting the stimulation parameters for the excitation of specific groups of fibres. Besides evoked activity, the VSR technique has been successfully applied to analyse spontaneous neural activity from the L5 dorsal rootlet of a rat [47] and the cervical vagus nerve of a pig [48].

The VSR technique was already investigated in earthworms [22], frogs [21], rats [19], pig vagus [48], and the median [24] nerves. To date, however, no investigation of the technique has been performed in the pig ulnar nerve. Because of the similar morphology of the pig nerve to the human nerve, including fascicle number and nerve diameter comparable to the human [40], pigs can be used to develop and assess neural interfaces that closely resemble human-sizes interfaces. Additionally, the superficial location of the ulnar nerve is an advantage over deeper nerves, as its easy accessibility and surgical approach can reduce the risk of complications (e.g., infection, inflammation and allergies) and the time of recovery in both acute and chronic experiments. Finally, the ulnar nerve is an important target for the development of hand prostheses because of its innervation territory.

This paper shows that a single stimulus event is sufficient for obtaining the velocity profile, which is in line with previous studies using the VSR technique [19,21,24]. This characteristic of VSR has clear advantages over methods that require multiple stimuli to characterise neural activity (i.e., stimulus-triggered average), including reducing the risk of neural injury due to prolonged electrical stimulation and providing a suitable platform for online neural recording and analysis. Moreover, contrary to single point measurements, the VSR technique enables the differentiation of afferent from efferent signals by selecting positive or negative values for the artificial delays.

The effect of pulse duration has never been investigated using the VSR technique. The choice of pulse duration has great importance in optimising charge injection, reducing the risk of tissue damage and electrode corrosion, and extending battery life. Our results showed a significant effect in the excitation of the fast fibres, with a larger current needed in the case of using a pulse duration of 100 μs. In the other three levels (500 μs, 1000 μs, and 5000 μs), pulse duration had no effect in activating the nerve, indicating that a stimulus of 500 μs is on the horizontal asymptotic section of the strength–duration curve. Consequently, this study shows that increasing the pulse duration from 500 μs to 1000 μs and 5000 μs did not affect stimulating the fast group of nerve fibres. Therefore, the use of shorter pulse durations seems a more reasonable choice for a future assessment of the influence of pulse width on the nerve activation thresholds.

Sensory nerve fibres with different conduction velocity ranges, convey specific functions to the central nervous system. While the fast myelinated fibres (Aβ) are responsible for mediating the sensation of touch, pressure, and joint positions, the slower thinly myelinated Aδ fibres represent free nerve endings conducting painful stimuli related to pressure and temperature. Through the VSR technique, this paper shows that the cutaneous branch of the ulnar nerve contains both Aβ and Aδ fibres, while the motor branch contains only Aβ fibres. These branches are also expected to contain small unmyelinated C fibres with velocities between 0.5 and 2 m/s, responsible for carrying information of various painful stimuli (e.g., mechanical, thermal, and chemical). The reason for using long interphase delays was to be able to separate the responses of the primary and the secondary stimulation phases (which also elicited an eCAP response). Because of the range of C-fibre velocities, theoretically, C-fibre responses could appear in a large time window. However, C-fibre activity was not observed in the eCAP recordings, likely due to the smaller energy for the slower action potentials [15]. Recording C-fibres with extraneural interfaces remains a significant challenge; Castoro et al. quantified the excitation properties of the vagus nerve in 9 dogs, and yet, C-fibres were observed in only 1 experiment [49].

### 4.1. Variations across and within Experiments

In three animals, the presence of two dominant conduction velocities was observed, corresponding to Aβ fibres (35–75 m/s) and Aδ axons (5–35 m/s). The ability to differentiate between the two populations might have been affected by the small distance between the stimulation and recording electrode. Still, the detected velocities are in accordance with a previous study by Schuettler et al., who reported the presence of two fibre types in the median nerve of a pig: a fast fibre group (60 m/s) and a slow fibre population (30 m/s) [24]. In these three cases, the slow group of fibres was observed when the cutaneous branch of the ulnar nerve was stimulated. In contrast, the fast fibre group was observed in all animals and both ulnar nerve branches. For the cutaneous branch, the fast fibre group had a mean velocity of 55.6 m/s, while the slower fibre group had a mean propagation velocity of 21.1 m/s. Alternatively, for the motor branch, the mean propagation velocity was 64.0 m/s.

The results show a significant variation in the detected velocity across the experiments; for the fast fibre group, while animal 04 showed a mean velocity of 41.1 m/s for the cutaneous branch, animal 07 had a mean velocity of 66.4 m/s. Variations in the detected conduction velocity with the VSR technique were observed previously in the nerve of frogs in vitro; the authors argued that such discrepancies can be explained by the slow degradation of the nerve tissue and changes in electrolytes [21]. This is the first paper to investigate the VSR technique in multiple subjects in vivo; therefore, because of the well-documented relationship between fibre diameter and conduction velocity [50], our results suggest that a likely reason for this variability is the morphological difference between the nerves. Nevertheless, it remains to be tested to what extent the VSR technique reflects morphological characteristics of the nerve and histological analysis is needed to verify such features.

Intra-animal variability in the detected conduction velocity was also observed (Figure 3). A possible reason for this effect is that, even in the same fibre-type group, the axons sizes are not entirely homogeneous. Consequently, with smaller stimulation amplitudes, the larger and faster axons (and consequently with smaller thresholds) within the same fibre group are recruited. As the stimulation increases, the velocity can decrease with the addition of smaller and slower axons, generating a spread in the velocity profile.

### 4.2. Limitations and Technical Difficulties

One of the limitations of the current study was the small distance (approximately 2.5 cm) between stimulating and recording electrodes, which resulted in the presence of stimulation artefacts for three animals (i.e., the motor branch in animals 08 and 04 and cutaneous branch in animal 06). Using a potentiometer to balance the impedances of the outer rings of the stimulation electrodes significantly reduced the stimulation artefact contamination, allowing the recording of 15 out of 18 branches. For the recording cuff, a silicon sheet was used to minimise current leakage. Still, further improvements could be made, including adjusting for the impedance imbalances of the recording cuff and blanking the amplifiers. The authors considered placing the recording cuff more proximally (closer to the elbow) to increase the distance between the electrodes; however, such a procedure would result in more invasive surgery, requiring the excision of the flexor carpi ulnaris muscle for accessing the nerve. A more invasive surgery would limit the applicability of the results to chronic studies or studies in which the animal should be perturbed as little as possible. Furthermore, the impedance measurement of the electrodes was not performed. Similar impedances can improve the rejection of common-mode noise and increase the capability of differentiating the contribution of small fibres in the eCAP [51]. For chronic recordings, fluctuations in the impedance values are expected to occur in the first days post-implant due to changes in the bioelectric medium [52]. Finally, further research is needed to investigate how well the VSR technique represents morphological characteristics of the nerve.

## 5. Conclusions

This study reports the first application of the velocity selective recording technique to analyse electrically evoked neural signals from the ulnar nerve of multiple pigs in vivo. The results demonstrate that it is possible to discriminate fibre-types based on their respective conduction velocity with a single stimulation event and no averaging. The results show that the underlying axonal diameter distribution appears to be bimodal in the cutaneous branch and unimodal in the motor branch. Furthermore, the results provide information on the thresholds of the activation of different fibre-types; thus, this technique can be used as an online tool to assist in selecting the stimulation parameters for the excitation of specific groups of fibres and for probing the neural circuitry, as well as investigating peripheral nerve interfaces.

## Figures and Tables

**Table 1:** Single animal velocity distribution (mean ± standard deviation)—the asterisk (*) denotes the animal and branch in which stimulation artefact contamination was observed.

Animal	Cutaneous Branch Velocity (m/s)	Motor Branch Velocity (m/s)
01	52.3 ± 5.4	59.6 ± 4.3
02	59.4 ± 4.5	63.0 ± 7.0
03	56.3 ± 3.3	57.7 ± 2.0
04	42.5 ± 5.6	*
05	49.9 ± 3.6	61.0 ± 7.6
06	*	74.6 ± 3.6
07	66.6 ± 5.2	66.8 ± 8.4
08	57.1 ± 1.7	*
09	58.6 ± 2.2	62.8 ± 1.1

## Data Availability

The data of this study are available from the corresponding author, F.R.A., upon reasonable request.
